# In-vivo evaluation of silk fibroin small-diameter vascular grafts: state of art of preclinical studies and animal models

**DOI:** 10.3389/fsurg.2023.1090565

**Published:** 2023-05-26

**Authors:** Alberto Settembrini, Gianluca Buongiovanni, Piergiorgio Settembrini, Antonio Alessandrino, Giuliano Freddi, Giulia Vettor, Eugenio Martelli

**Affiliations:** ^1^ Department of Vascular Surgery, Foundation IRCCS Ca’ Granda Ospedale Maggiore Policlinico, Milan, Italy; ^2^ Postgraduate School of Vascular Surgery, Università Degli Studi di Milano, Milan, Italy; ^3^ Department of Biomedical and Clinical Sciences, L. Sacco University Hospital, Università degli Studi di Milano, Milan, Italy; ^4^ Silk Biomaterials Srl, Lomazzo, Italy; ^5^ Heart Rhythm Center, Department of Clinical Electrophysiology and Cardiac Pacing, IRCCS Monzino Cardiology Center, Milan, Italy; ^6^ Department of General and Specialist Surgery, Sapienza University of Rome, Rome, Italy; ^7^ Saint Camillus International University of Health Sciences, Rome, Italy; ^8^ Division of Vascular Surgery, S. Anna and S. Sebastiano Hospital, Caserta, Italy

**Keywords:** silk fibroin, vascular graft, animal models, biological graft, preclinical *in vivo* studies

## Abstract

Autologous vein and artery remains the first choice for vascular grafting procedures in small-diameter vessels such as coronary and lower limb districts. Unfortunately, these vessels are often found to be unsuitable in atherosclerotic patients due to the presence of calcifications or to insufficient size. Synthetic grafts composed of materials such as expanded polytetrafluoroethylene (ePTFE) are frequently employed as second choice, because of their widespread availability and success in the reconstruction of larger arteries. However, ePTFE grafts with small diameter are plagued by poor patency rates due to surface thrombogenicity and intimal hyperplasia, caused by the bioinertness of the synthetic material and aggravated by low flow conditions. Several bioresorbable and biodegradable polymers have been developed and tested to exploit such issues for their potential stimulation to endothelialization and cell infiltration. Among these, silk fibroin (SF) has shown promising pre-clinical results as material for small-diameter vascular grafts (SDVGs) because of its favorable mechanical and biological properties. A putative advantage in graft infection in comparison with synthetic materials is plausible, although it remains to be demonstrated. Our literature review will focus on the performance of SF-SDVGs *in vivo*, as evaluated by studies performing vascular anastomosis and interposition procedures, within small and large animal models and different arterial districts. Efficiency under conditions that more accurately mime the human body will provide encouraging evidence towards future clinical applications.

## 1. Introduction

Bioengineering is becoming a crucial aid to cardiovascular surgery, especially with the introduction of endovascular means. Therefore, knowledge of the behavior of surgical or endovascular grafts and their long-term impacts onto large and small vessels is vital to the cardiovascular specialist.

This scientific approach improved the management of vascular pathologies through different techniques and materials. A constant dialogue between clinicians and bioengineers is essential for the development new effective graft materials, with desirable characteristics over the aforementioned issues.

Cardiovascular diseases are projected to grow in the following years to an estimated overall mortality of 23.4 million in 2030 (1). Moreover, the age of patients who require treatment has been increasing. Vascular and cardiac surgery are the eminent fields for surgical or endovascular treatment of both large and small vessels. Most cardiovascular diseases are caused by the occlusion or narrowing of arteries of medium or small diameters and in this scenario, autologous grafts are currently the gold standard for the revascularization of coronary and lower limb arteries (2).

However, the use of these conduits is often limited because atherosclerosis is a systemic disease that can involve all arterial districts, including arterial conduits most harvested for revascularization. Another issue arises in patients who underwent a prior harvesting of an arterial or venous conduit, limiting the availability of a possible graft for further interventions.

Synthetic grafts provide a viable solution for large vascular replacement, but their performance with low flow conditions in smaller vessels is disappointing. For this reason, vascular tissue engineering aims to find a solution to have a functional and infection-resistant graft to replace the diseased artery. We consider the need for endovascular but also open revascularization and look for available and safe alternative materials applicable to all the small vessels requiring intervention.

Indeed, the most critical problem is to obtain suitable materials for small grafts assuring a long-term patency and infection resistance.

About the first aspect, vascular and cardiac surgeons are skilled in the harvest of autologous vessels, proven to maintain patency with low thrombogenicity. The most employed vessels for open surgical revascularization include mammary or radial arteries, or different veins of the superficial system of the upper and lower limbs, (cephalic, basilic, great and small saphenous veins). In surgery of peripheral arterial disease, revascularization by means of in-situ venous conduits is also used to bypass occlusions of the distal arteries of the limbs.

Secondly, to prevent infection, autologous grafts should be preferred over synthetic ones, particularly in case of revascularization of wet gangrene of the lower limbs.

Different authors are reporting experiences of using silk fibroin (SF) *in vivo* in anima (for the most small) models in a preclinical phase as an arterial substitute and its further development, in terms of patency, endotelialization, low restenosis, surrounding tissues involvement, and resistance to infections.

*Silk* is a natural, versatile protein biopolymer which is produced by various insects (silkworms, spiders, scorpions, mites, and flies). This ancient textile material has long been used to prepare sutures in surgery thanks to its biocompatibility and remarkable tensile strength (3). Among the variants, a regenerated silk fibroin obtained from Bombyx mori silkworm cocoons is widely explored for regenerative medicine.

Silk fibroin shows suitable properties allowing its use for vascular applications: biocompatibility, tunable biodegradation, low immunogenicity, ability to adapt to different geometries and preparations (4, 5), mechanical strength, easy accessibility, cost-effectiveness, and easy green processing (6). As a biodegradable the starting material can be easily purified and processed in different 2D/3D shapes. It is not immunogenic in humans and favors angiogenesis, an essential feature for tissue repair and regeneration (7, 8).

**Figure 1:**
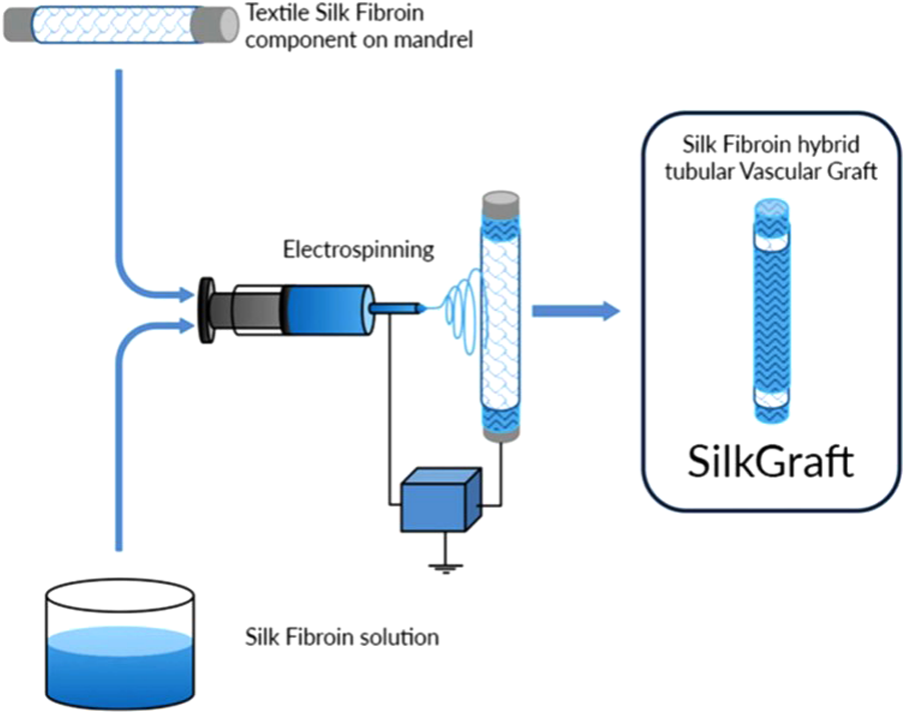
Description of creation process of silk fibroing vascular graft through hybrid-textile electrospun architecture.

**Figure 2:**
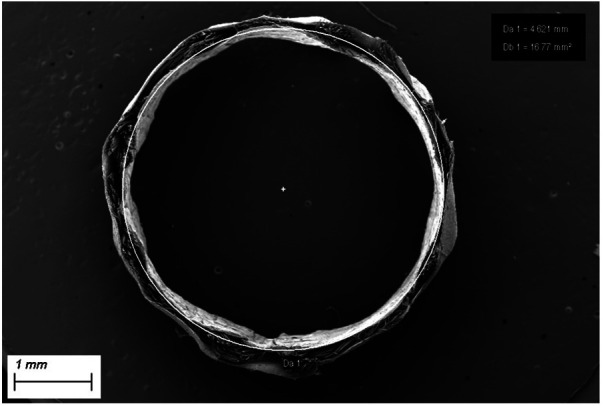
Cross section of SF-SDVG showing the tri-layered wall with the two electrospun layers encasing the intermediate textile layer.

These results are possible because electrospinning has the ability to mimic the nanoscale properties of fibrous components (collagen and elastin fibrils) of the extracellular matrix and to realize a range of biochemical, topographical, and mechanical properties conducive to improved cell interactions (9) (Figures 1, 2).

Our review aims to provide a comprehensive overview of the principal fields of preclinical use of silk fibroin as a vascular substitute in different animal models evaluating silk fibroin (SF) behaviour in term of endothelialization, biocompatibility and short and long term patency.

## 2. Aim, search protocol and selected studies

Although other reviews on tissue-engineered vascular grafts have been previously published (10), including regarding specific applications of silk biomaterials (11), the aim of the present research is to collect and report the studies in which SF-based small-diameter vascular grafts were evaluated *in vivo* by performing vascular anastomosis and interposition procedures within small and large animal models and different arterial districts.

The Preferred Reporting Items for Systematic Reviews and Meta-Analysis Protocols (PRISMA-P) statement was used to obtain a strict paper selection (12).The PubMed, Scopus and Embase databases were questioned to identify studies, without specifying an interval of publication years. All types of articles were initially included, whereas review articles were then manually discarded following the selection algorithm.

To perform the initial review process, based on paper and abstract title search, the combination of keywords used, connecting Boolean operators, was:

((silk fibroin) AND (vascular)) AND (*in vivo*)

The Rayyan QCRI [Qatar Computing Research, Doha, Qatar (available at https://rayyan.qcri.org/)] was chosen to manage and screen selected papers. After the collection of all search results, duplicates were automatically excluded before the screening process.

A series of exclusion criteria were considered to select the articles that best applied to our objectives. Articles and abstracts not containing the selected keywords in their title were excluded. Subsequently, the remaining articles were screened for relevance of topics according to the aims of our review, as well as pertinence of the experimental model in which SF was evaluated. Indeed, studies exclusively including *in vitro* analysis of the biological response to silk-based biomaterials were not applicable to our review process. Furthermore, out of the studies where SF was applied *in vivo*, those where the grafting biomaterial was not inserted by vascular anastomosis (e.g., in case of subcutaneous implantation) were also excluded. Finally, remaining articles utilizing vascular grafts developed from materials not including SF were discarded.

As a result of this selection process, a total of 31 articles concerning *in vivo* assessment of SF-based vascular grafts were reviewed, with experimental models ranging from small to large animals: murine, leporine, canine, porcine and ovine. Henceforth, the included studies are presented according to the respective animal model. Because the results were not fit for a meta-analytic investigation due to the heterogeneity of the *in vivo* animal models and graft materials, they were discussed in a narrative way.

**Figure 3:**
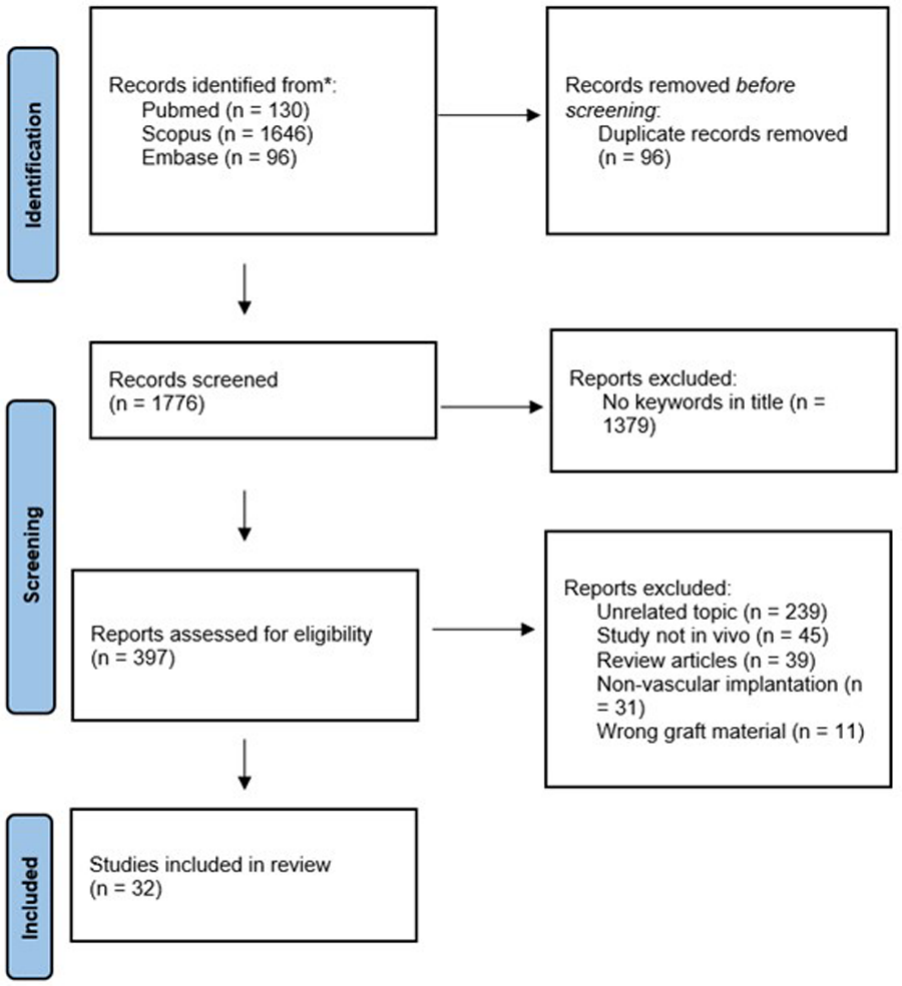
The flow chart for the identification and selection of studies for the review via databases and registers.

The flow chart for the selection of studies is shown Figure 3.

## 3. SF in small animal models

Compared to larger animals, small animals are easier to handle and less expensive. However, the technical difficulty of their size makes the surgical insertion of vascular conduits difficult. Rats and rabbits are the most employed, and both can often receive conduits with very small (2 to 5 mm) and small (2 mm) diameters (13). Accordingly, most of the published experimentation of SF grafts *in vivo* was performed on murine and leporine models.

### 3.1. SF in murine models

Some authors discourage the use of murine models to test the patency of short graft portions, especially in the absence of a clinically relevant control (13). Although size-mismatched grafts up to 4 mm in diameter have been employed, the small size of murine arterial conduits often restricts their utility for the assessment of grafts to the aortic (1 to 2 mm diameter) or femoral (1 mm diameter) sites. The main issue with using rats to simulate human biocompatibility to very small conduits is that these animals typically have high patency rates for ePTFE grafts, unreflective of the clinical outcomes of such material.

Some authors report that the surgical interposition of completely silk-based vascular grafts in mice is often coupled with *in vitro* demonstration of rapid endothelial cell attachment and excellent resistance to blood clot and fibrin network formation suggesting that silk fibroin might be a promising material to develop vascular prostheses for smaller arteries (14). Electrospun silk conduits have been shown to enhance biocompatibility (15). *In vitro*, they enhanced endothelial cell proliferation 5-fold, from day 1 to 6, significantly greater than ePTFE controls. Moreover, they demonstrated comparable elasticity to rat aorta and were significantly more elastic than ePTFE. When implanted into the abdominal aorta of rats, silk conduits were well tolerated up to 24 weeks, showing only low levels of inflammatory cells and neointimal hyperplasia, and demonstrated complete patency at all time points when compared to control animals implanted with commercial ePTFE of similar diameter (15). Overall survival over 24 weeks was 95% for the silk grafts, while ePTFE failure occurred at 27%, with graft failures relating to deaths occurring post-operatively or, alternatively, due to complete occlusion. These results demonstrate a high degree of variability in ePTFE survival, which is consistent in observations in humans, and were related to rapid coverage of endothelial cells upon the electrospun silk graft lumen, contrary to ePTFE, which remained largely uncovered, even at 24 weeks (15).

Other studies produced small caliber fibroin matrices with electrospinning techniques with the aim to replace small arterial segments. A study evaluating electrospun fibroin scaffolds implanted into the rat abdominal aorta concluded that formation of vascular tissue containing elastin occur already at 7 days after implantation on fibroin scaffold without prior *in vitro* cellularization (16). Indeed, fibroin matrices not only allowed host cell infiltration and extracellular matrix remodeling, but also the formation of vasa-vasorum in the outer layer of the fibroin material.

A different study evaluated the impact of porosity of gel-spun silk tubes upon their biological features *in vivo*, including degradation of the biomaterial, cellularity, and host integration over time (13). Tubes with higher porosities showed early improvements in cell colonization that progressively increased over time. However, none of the highly porous tubes remained patent at 6 months, likely because the remodeling can induce bulk mechanical failure or a compromised blood-material interface (5).

The introduction of functional peptides is useful for improving the mechanical properties of SF. In the last decade, germ line transformation of silkworms was developed using transposons to obtain fusion of exogenous proteins with fibroin. Genes encoding the exogenous protein of interest may be injected into the eggs of silkworm so that the transgenic silk product will be produced in the worm's silk gland and expelled in the cocoon silk.

A study evaluated the performance of SDVGs obtained by four kinds of recombinant SFs derived from the fusion of peptide sequences from laminin B1 and fibronectin (17). Despite a slight decrease in tensile strength, compared with native SF, the adhesive activities of mouse endothelial and smooth muscle cells have been shown to increase significantly with recombinant SF films. When implanted into the rat abdominal aorta *in vivo*, grafts coated with recombinant SF allowed longer migration distance of the endothelial cells from the anastomotic margin. In view of these results, recombinant SF incorporating laminin peptide sequence can be potentially used as a vascular graft material.

In a different study, vascular endothelial growth factor (VEGF) introduced into the SF heavy chain to improve its properties showed greater enhancement of cellularization behavior compared to wild type (WT)-SF (18). VEGF-SF also showed lower platelet adhesion than the RGD SF and WT-SF. *In vivo*, early endothelialization was observed for VEGF transgenic SF, including the occurrence of native tissue organization at three months after implantation in rat abdominal aorta.

Moreover, VEGF-SF has been shown to support significantly higher and earlier endothelialization in the central part of aortic grafts in mice compared to WT-SF (19). However, complete endothelialization was not confirmed after 3 months of implantation in neither of the fibroin grafts.

*In vivo* substitution of arterial segments with SF grafts has also been attempted with carotid arteries in murine models. In one of such models, a small-sized graft of 0.9 mm inner diameter was braided from a SF thread and interposed to carotid arteries in mice using a cuff technique (20). Upon histological analysis after graft harvesting, endothelial cells had already started to proliferate at 2 weeks after implantation while, after 4 weeks, the luminal surface was found to be covered with a neointimal layer. Graft patency was confirmed at up to 6 months after implantation.

In a different model employing patches of SF with a gelatin hydrogel incorporating simvastatin micelles for sustained release of simvastatin was investigated for its promotional effects on endothelial progenitor cell mobilization from bone marrow and recruitment to sites of vascular injury, exhibiting acceleration of re-endothelialization (21). *In vivo* implantation of the patches incorporating simvastatin significantly increased the recruitment of circulating endothelial progenitor cells (EPCs) and allowed complete re-endothelialization on the SF patches at 2 weeks after implantation in rat carotid arteries.

A significant number of studies employing SF *in vivo* into small animal models do so by combining it with other synthetic graft materials to obtain different mechanical and physiological outcomes, especially by developing a SF coating of the inner lumen. Indeed, coating is an important factor to maintain the strength of the anastomotic region of vascular grafts, and to prevent the blood leak from the vascular grafts after implantation.

A study evaluating electrospun poly (L-lactic caprolactone) /SF small-diameter grafts, loaded with VEGF and heparin, found that such artificial blood vessels have good cytocompatibility and histocompatibility, but the patency and degradability need to be improved (22). Indeed, laser Doppler perfusion imaging showed that the blood flow velocity decreased at 1 day after implantation of the artificial blood vessel, and then gradually decreased, until the third week. Moreover, despite new angiogenesis and uniform endodermis were formed on the inner wall of the sample, scanning electron microscopy showed that the fibers were basically broken at the 6th week of implantation of the artificial blood vessel, and the fibers became thinner.

In an alternative combination, a tri-layered nano-fiber scaffold of SF and poly-caprolactone (PCL), fabricated using a sequential electrospinning method and containing Spirulina extract in its inner SF layer, was implanted into rat carotid artery and evaluated for histological analysis after 3 weeks (23). Besides excellent mechanical properties *in vivo* (longitudinal and circumferential tensile strength, burst pressure strength, and suture retention strength), vessel patency was maintained, and the inner lumen of the scaffold showed regenerated endothelial cells. Platelet adhesion was supposedly countered by the presence of Spirulina extract in the inner SF layer.

A different research group developed a SF-coated poly(ethylene terephthalate) (PET) graft of diameter <6 mm. The tubular PET graft was produced through the double-raschel knitting technology and then coated with porous SF prepared using glycerin (Glyc) as porogen. *In vivo* outcomes of SF(Glyc)-PET graft were compared with a widely used commercial gelatin-coated PET graft (24). In implantation experiments in rats, the SF(Glyc)-coated PET graft was rapidly degraded *in vivo* and remodeling to self-tissues was promoted compared with the gelatin-coated PET graft. Importantly, unlike the gelatin-coated PET graft, side reactions such as thrombus formation and intimal hyperplasia were not observed in the SF(Glyc)-coated PET graft.

In a parallel study SF and PET tubular grafts of 1.5 mm diameter, produced with a double-raschel knitting machine, were coated with aqueous SF or gelatin (G) to make four types of vascular grafts (SF/SF, SF/G, PET/SF, and PET/G, shown as “base/coating material,” respectively) (25). The four types of grafts were implanted into rat abdominal aortae (*n* = 6, respectively) and explanted 2 weeks or 3 months later. Two weeks after implantation, no significant differences were found among the kinds of grafts in biological reactions evaluated by histopathologic examination. However, a remarkable difference was observed after 3 months in terms of area of tissue infiltration into the graft wall, 2.5 times larger in SF/SF than that in PET/G. The endothelialization was achieved almost 100% in SF/SF, despite only 50% was achieved in PET/G. Therefore, SF delivered promising results both as base and as coating materials for small-diameter vascular prostheses.

Another study of the same group focused on the coating of 1.5 mm diameter SF grafts produced by double-raschel knitting with SF solutions at different concentrations (1%, 2.5%. 5%, and 7.5%) containing polyethylene glycol diglycidyl ether (PGDE) as cross-linker. The effect the different coatings on tissue infiltration and remodeling was investigated (26). The grafts were implanted in the rat abdominal aorta and removed after 3 weeks or 3 months. While SF concentration had no significant effects on the patency rate, 2.5% SF coating was found to be the most suitable concentration, based on the characteristics of less stenosis, early tissue infiltration, and less neointimal hyperplasia.

In the work reported in a distinct study, small-diameter vascular grafts 1.5 mm in diameter were prepared by coating a double-raschel knitted silk fiber graft with SF aqueous solution containing PGDE as a cross-linking agent (27). Eight weeks after implantation of the grafts in rat abdominal aorta, early formation of thrombosis was avoided possibly as an effect of the SF coating, providing also protection against leakage of blood from the graft, and elasticity to the graft.

As an alternative application, a blend of SF and thermoplastic polyurethane (PU, Pellethane®) was employed in order to produce by electrospinning a cardiovascular patch with appropriate elastomeric characteristics without sacrificing the excellent tissue affinity and biocompatibility of silk (28). Upon implantation into rat abdominal aorta, histological evaluation revealed that with increasing SF content the tensile strength and elasticity of the patch decreased, while tissue infiltration, elastogenesis and endothelialization were shown to improve. Indeed, the authors propose the blended patch as an attractive alternative material that could induce the growth of a neo-artery composed of tissue present in native artery.

SF has also been applied as substrate for cylinder stents of small diameter (29). In a single study, a solution of native SF directly taken from the silk gland was used to prepare a cylindrical stents of 1 mm diameter and 5 mm length. The stents were inserted *in vivo* into the abdominal aorta of 22 rats and fixed using methylmethacrylate. Such technique of stent anastomosis produced significantly shorter ischemia time during implantation compared to conventional sutures and, after 4 months, the anastomosis was shown to be functionally patent in all cases. However, thrombus formation, frequent and severe abdominal infections, and heavy host rejection remain critical issues.

### 3.2. SF in leporine models

Rabbits are the small animal of choice for conduits 1 to 4 mm in diameter, enabling bilateral implantation of longer conduits and having a greater similarity than rats to humans in coagulation, endothelialization, and patency. Carotid artery grafting is convenient in the rabbit due to technical feasibility, similar longitudinal tension to humans, as well as comparable endothelial response to prosthetic conduits. In fact, patency rate over time closely simulates the clinical response to chronic ePTFE grafts seen in humans.

A study employed a 3 mm diameter tubular scaffold, produced by braiding SF yarns and coating with a SF-PGDE aqueous solution, to evaluate endothelialization and steady-state blood flow *in vivo* by implanting and replacing a common carotid artery in rabbits (30). Doppler ultrasound and angiography demonstrated graft patency without aneurysmal dilations or significant stenoses at any time point, with waveforms comparable to the native contralateral artery. In addition, immunohistochemistry results showed that a clear and discontinuous endodermis appeared after one month of implantation, while a full endothelial layer covered the inner surface of the graft after three months. This finding was backed by the RT-PCR results indicating that the gene expression level of CD31 of cells populating the SF graft was 45.8% and 75.3% by that of the contralateral carotid artery at 3 months and 12 months respectively, while the VEGF gene showed a high expression level that continued to increase after implantation.

For what concerns SF blends with other polymers as vascular grafts in leporine models, a study implemented electrospun bi-layered scaffolds made of an outer layer of SF-poly(L-lactide-co-*ε*-caprolactone) (SF/PLCL) and an inner layer obtained by shell-core electrospinning, in which the shell was SF/PLCL or PLCL alone and the core was a 15% aqueous heparin (Hep) solution, yielding SF/PLCL/Hep or PLCL/Hep grafts (31). A rabbit carotid artery replacement model was used to evaluate the vascular scaffolds *in vivo* (1.5 cm in length, 2 mm in diameter). Compared to the graft with PLCL/Hep as inner membrane, the hydrophilicity of the graft including SF (SF/PLCL/Hep) was significantly improved, with optimal biocompatibility and maintained lumen patency for 3 months after carotid artery transplantation at ultrasound examination.

SF has also been utilized to enhance the biocompatibility and hemocompatibility of commercially available 4 mm ePTFE grafts (32). A film of SF has been formed onto the inner surface of the ePTFE graft and then it has been exposed to sulfur dioxide plasma to obtain sulfonated SF. The study compared the patency rate between 12 of such SF-modified grafts and 10 unmodified ePTFE grafts used to replace a section of the lower abdominal aortic artery in rabbits. The patency rates of SF-modified ePTFE grafts were found to be higher than those for the unmodified grafts at all timepoints (on day 3, from day 4 to 3 months, and at 3 months the patency rates were 100% vs. 60.0%, 91.7% vs. 33.3%, and 91.7% vs. 20.0%, respectively). Moreover, grafts harvested 3 months postoperatively showed that approximately 84% of the inner surface of the SF-modified grafts were covered by endothelial cells, compared to 11% of the inner lumen of the unmodified grafts, also harboring an inflammatory and thrombogenic substrate of activated platelets, erythrocytes, and newly formed extracellular matrix.

## 4. SF small diameter vascular grafts in large animal models

### 4.1. SF in canine models

Most of the published research performing arterial anastomosis of SF-SDVGs *in vivo* involved murine models, in which patency and remodeling potential were generally evaluated upon arterial grafting of the abdominal aorta. For this reason, the transition of such *in vivo* experiments towards larger animal models might be able to provide more ideal empirical conditions. According to the retrieved literature, canine models were the second-most employed animal for experimental purposes involving SF after the abovementioned murine models. However, some authors have shown that dogs are more susceptible to blood clotting than other animal species, thereby introducing a possible bias on the long-term patency rates of the implanted SF-SDVG in some settings (13). Furthermore, research contradicting the assumptions of low patency and absence of spontaneous endothelialization attributed to prosthetic vascular conduits implanted in dogs no longer support the use of canine models in the preclinical assessment of arterial grafts (33). Although originally advised, compared to other animals, the canine model has more differences and variability in hemostatic processes, as well as a proclivity for quick endothelialization of vascular prostheses.

Some studies have tested the features of hybrid combinations of SF and different synthetic graft materials to achieve improvements in mechanical and biological characteristics. SF has been used as biological sealant to counter thrombogenesis and foreign body or inflammatory reactions of textile arterial prosthesis (25). Six SF-coated polyester (PET) velour vascular grafts were implanted in the abdominal aorta of dogs for scheduled periods and outcomes were compared with commercial collagen-impregnated grafts and untreated external velour grafts. Besides rendering the graft impermeable to blood, the SF coating was shown to improve endothelialization of the synthetic graft material by favoring the outgrowth of a neointimal layer (34).

A different application in a canine model evaluated the mechanical properties and tissue biocompatibility of a surgical patch made by electrospinning a blend of SF and thermoplastic polyurethane by replacing part of the wall of the canine descending aorta (35). After 3 months the histological examination revealed excellent intimal tissue coverage of the intraluminal surface, absence of calcium deposition, and minimal inflammatory reaction, without signs of degradation.

Regarding the application of SF grafts in canine carotid arteries, a study monitored the patency of knitted SF grafts, double-coated with 5% SF and 10% PGDE, by means of Doppler sonography and histological analysis (36). Of five implanted grafts, 3 mm in diameter and 3 cm length, four were observed over four weeks and one graft was observed over one year. Indeed, while no significant changes were observed on the short term, hemodynamic alterations were observed after one year, suggesting intimal plaque or stenosis formation on behalf of the middle and proximal portion of the vascular prosthesis despite its patency. Moreover, albeit SF was almost degraded and replaced by fibrous tissue without any sign of necrosis, calcification, or infection, endothelialization of the graft appeared as incomplete.

In another study the same group compared the outcomes of thirty-five SF-coated SF grafts and five ePTFE grafts implanted via end-to-end anastomosis into the carotid arteries of dogs, determining a non-significant difference in patency rates at six months of 7.8% and 0%, respectively (37). However, the SF grafts were found to induce unique histological responses, such as maintaining the thickness of the luminal layers despite SF fiber degradation, due to fibrin accumulation and collagen fiber replacement with endothelialization at three months post-implantation.

Canine femoral arteries were also subjected to surgical replacement with SF grafts. A study aiming to investigate the biocompatibility and function of chitosan-sulfated SF prostheses randomly allotted eight dogs towards an experimental group in which the graft was implanted and a control group which received no intervention (38). Six months after implantation, the architecture of endothelial and smooth muscle cells and coagulation function were found to be comparable between the two groups.

In a recent study, knitted SF grafts coated with SF-Glycerin were implanted in the femoral artery of six dogs and patency was assessed periodically with Doppler sonography and histologically analyzed (39). Five of the six grafts exhibited a high patency rate, with evidence of endothelialization in the central part of the graft as as three months post-implantation, with no luminal narrowing. In the remaining case, occlusion was secondary to progressive graft bending and consequent thrombosis. For this reason, the authors suggest that enhancing biodegradation and early endothelialization of the graft may counter the onset of thrombosis due to mechanical factors.

### 4.2. SF in porcine vs. ovine models

**Figure 4:**
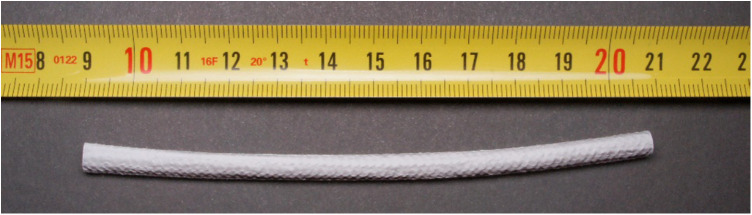
Silk fibroin-based small diameter vascular graft (SF-SDVG) of 12 cm length and 4.5 mm inner diameter ready for the implantation.

Sheep are indicated as the large animal model of choice for vascular conduits with diameters of 4 to 6 mm (13) (Figure 4). Porcine models are also frequently employed for assessing vascular conduits, with platelet activity and coagulation like humans, as well as a proclivity for biomaterial implant calcification. However, complete endothelialization was shown to occur within three months post-implantation in all polyester conduits in pigs, whereas only peri-anastomotic endothelialization was observed in sheep, which more closely matches human physiology.

The performance of SF in a pig model was evaluated by employing a multilayered silk protein coating for drug-eluting stent systems (40). In this study, the SF layer was intended as a drug carrier and delivery system of molecules such as heparin, paclitaxel, and clopidogrel to modulate vascular cell responses to stent placement. The preliminary short term (2.5 h) *in vivo* study in a porcine aorta showed integrity of the silk coatings after implantation and the reduction of platelet adhesion on the heparin-loaded silk coatings.

In a more recent study, a hybrid vascular using SF and polyurethane (Silkothane), manufactured by electrospinning, was employed to evaluate patency and short-term remodeling in a sheep model of arteriovenous shunt (41). Nine Silkothane grafts were implanted between the common carotid artery and the external jugular vein of sheep and assessed at 30, 60, and 90 days. Eight of nine sheep (89%) showed complete primary unassisted patency of the graft at the respective time of sacrifice (one case of surgery-related thrombosis excluded), while microscopic analysis found coverage by endothelial and inflammatory cells and pseudointimal formation inside the graft lumen, especially at the venous anastomosis, with no impairment of the functionality of the shunt.

**Figure 5:**
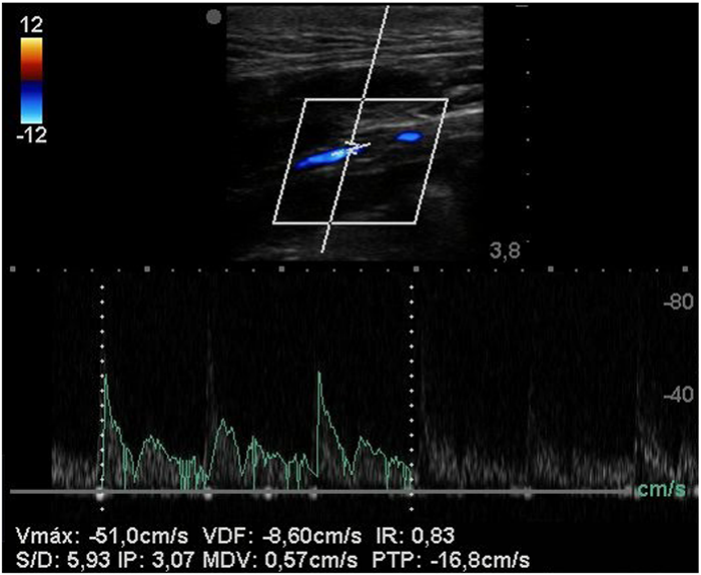
Duplex ultrasound of SF-SDVG in the carotid of a minipig after 1 month shows regular patency without any turbulence or velocity acceleration.

**Figure 6:**
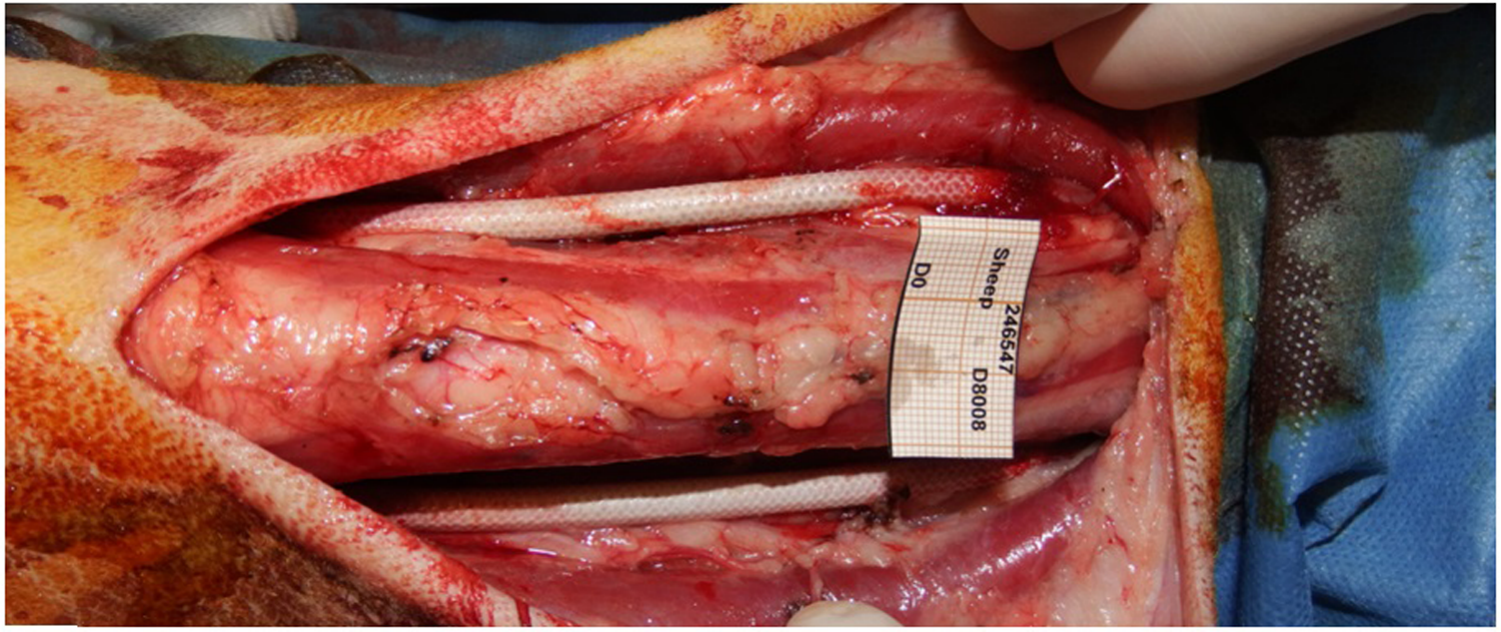
SF-SDVGs correctly implanted as vascular substitute of common carotid artery of both sides.

Some authors initiated pilot studies to assess whether the porcine or the ovine animal model would be better to use for future trials regarding the suitability of the surgical model and the long-term biological outcomes of interposed SF-SDVGs. In a single study, the carotid arteries of 1 minipig and 1 sheep were replaced with novel a tri-layered SF graft, monitored with Doppler sonography and angiography, and histopathology of explants was examined after four weeks (42) (Figure 5). Carotid implantation was preferred over femoral arteries to mimic clinical use and to avoid anatomical limitations. In both animal models the grafts were found to be patent, without any evidence of aneurysm, dilation, dissection, blood collection or signs of infection (Figure 6).

While in the mini-pig endothelial-like cells were found along the whole surface of the graft neointimal layer, endothelialization within the grafted sheep carotids was restricted to peri-anastomotic sites. This reflects a slower rate of endothelialization in the ovine model, closer to what occurs in humans. Microscopic evidence of an ongoing foreign body inflammatory reaction was observed in both animals, but only in the ovine model no concurring subendothelial hyperplasia and no damage to neighboring tissues had occurred. Therefore, in light of the biological outcomes observed, as well as several technical and practical advantages, the ovine model was deemed more suitable than the minipig for long-term studies aimed at assessing the performance of SF grafts. According to the findings of such pilot study in large animals, the use of a small caliber vascular graft entirely made of pure SF has been validated by favorable biologic outcomes coupled to its simplicity in handling and surgical feasibility and, as a result, can be designed as an “off-the-shelf” device meant for preliminary clinical trials on humans.

Table 1 summarizes all the studies in literature about the SF animal models.

**Table 1:** Summary of the studies of *in vivo* use of silk fibroin grafts in the different animal models.

Graft composition	Graft inner diameter	Animal model	Implantation site	Implantation time	Author, year
Braided SF	1.5 mm	Sprague-Dawley Rats	Aorta	18 months	Enomoto et al., 2010
Electrospun SF	1.5 mm	Sprague-Dawley Rats	Aorta	3, 6, 12, 24 weeks	Filipe et al., 2018
Electrospun SF	1.5 mm	Lewis Rats	Aorta	1 week	Cattaneo et al., 2013
Gel-spun SF	1.0–1.5 mm	Sprague-Dawley Rats	Aorta	1, 3, 6 months	Rodriguez et al., 2019
Double Raschel-knitted TG-SF	1.5 mm	Sprague-Dawley Rats	Aorta	2 weeks	Asakura et al., 2014
Double Raschel-knitted TG-SF	1.5 mm	Sprague-Dawley Rats	Aorta	2, 4, 8 weeks	Saotome et al., 2015
Computer-controlled braided TG-SF	1.5 mm	Sprague-Dawley Rats	Aorta	2 weeks; 3 months	Fukuyama et al., 2017
Braided SF	0.9 mm	C57BL/6 Mice	Carotid Artery	1, 2, 4 weeks; 3, 6 months	Tanaka et al., 2020
Dip-coated SF/gelatin patches + Simvastatin micelles	N/A	F344 Rats	Carotid Artery	2 weeks	Thitiwuthikiat et al., 2015
Electrospun PCL/SF + VEGF + Heparin	1 mm	Rats	Carotid Artery	3, 4, 6 weeks	Yue et al., 2022
Electrospun PCL/SF + Spirulina	*	Rats	Carotid Artery	3 weeks	Kim et al., 2015
Double Raschel-knitted PET + SF(Glyc) Coating	1.5 mm	Sprague-Dawley Rats	Aorta	2 weeks; 3 months	Tanaka et al., 2020
Double Raschel-knitted SF + SF Coating	1.5 mm	Sprague-Dawley Rats	Aorta	2 weeks; 3 months	Fukuyama et al., 2015
Double Raschel-knitted SF + SF/PDGE Coating	1.5 mm	Sprague-Dawley Rats	Aorta	3 weeks; 3 months	Fukuyama et al., 2015
Double Raschel-knitted SF + SF Coating	1.5 mm	Sprague-Dawley Rats	Aorta	2, 8 weeks	Yagi et al., 2011
Electrospun SF/PU Patches	N/A	Sprague-Dawley Rats	Aorta	1, 3, 6 months	Chantawong et al., 2017
Dip-coated SF Stents	1 mm	Sprague-Dawley Rats	Aorta	16 weeks	Smeets et al., 2016
Braided SF + SF Coating	3 mm	NZ White Rabbits	Carotid Artery	2 weeks; 2, 3, 6, 12 months	Li et al., 2019
Electrospun PLCL/SF + Heparin	2 mm	NZ White Rabbits	Carotid Artery	2 weeks; 1, 2, 3 months	Jin et al., 2019
SF-coated ePTFE	4 mm	NZ White Rabbits	Aorta	3 months	Zhang et al., 2017
SF-impregnated Dacron	8 mm	Mongrel Dogs	Aorta	4 h; 3 days; 2 weeks; 1, 3, 6 months	Huang et al., 2008
Electrospun SF/TPU Patches	N/A	Beagle Dogs	Aorta	3 months	Shimada et al., 2017
Double Raschel-knitted SF + SF Coating	3 mm	Beagle Dogs	Carotid Artery	4 weeks; 12 months	Aytemitz et al., 2013
Double Raschel-knitted SF + SF Coating	3.5 mm	Beagle Dogs	Carotid Artery	1, 2, 3 days; 1, 2, 3 weeks; 1–7 months	Haga et al., 2017
Chitosan-sulfated SF	*	Beagle Dogs	Femoral Artery	6 months	Ma et al., 2017
Double Raschel-knitted SF + SF Coating	3.5 mm	Beagle Dogs	Femoral Artery	3, 5, 12 months	Tanaka et al., 2021
SF/Heparin/Clopidrogrel-coated Metallic Stents	6 mm	Yorkshire Swine	Aorta	2.5 h	Wang et al., 2008
Electrospun SF/PCU	6 mm	Sheep	Carotid-Jugular AV Shunt	1, 2, 3 months	Riboldi et al., 2020
Electrospun SF/TEX	5 mm	Sheep and Minipig	Carotid Artery	1 month	Alessandrino et al., 2019

* Not present in the paper.

## 5. Discussion

While synthetic grafts (including PET or ePTFE) are a viable option in the replacement of large vessels, these have been shown to perform poorly when replacing small diameter vessels. Consequently, the need for an effective non-autologous vascular graft material for districts such as coronary and peripheral arteries appear to be still an open task for surgeons and bioengineers.

The optimal vascular graft should have scaffolds that mold the graft with an adhesive matrix primarily made of fibrin and vascular cells. Such biomaterials should also be capable of replacing the host's tissue, have a composition that is strong enough to prevent rupture, and have a sufficient size for clinical use.

The benefit of employing natural polymers is their built-in bioactivity, which results in favorable characteristics for graft regeneration.

Silk shows some properties that make it suitable to be a substrate for the creation of vascular grafts or in general vascular scaffolds for the healing of endothelial cells. They could be considered in two different groups: mechanical property and on the other hand biological properties as immune response and its interaction with vascular cells, biodegradation and haemocompatibility.

Multidisciplinary scientific efforts have converged to pinpoint the advantageous characteristics of vascular scaffolds in the quest to increase the clinical viability of vascular grafts. Indeed, the physical and chemical characteristics of the vascular scaffolds pragmatically dictate their therapeutic outcomes. Additionally, by adjusting the graft's characteristics, it is possible to calibrate the remodeling and formation of a neo-intimal layer in an implanted vessel. The ability to attract and recruit host cells while also providing them with a favorable microenvironment for growth, hemocompatibility, and minimal immunogenicity are just a few of the desirable features from an ideal vascular biomaterial.

Recruiting and attracting host cells is the base of endothelialization and it is a critical point in such a research especiallyfacing potentially biologic scaffolds. Endothelialization might be defined as cells and smooth muscle cells migrated into the fibroin graft early after implantation becoming organized into an endothelium and a media-like smooth muscle layer14. More specifically a transanastomotic endothelialization consists in the growth of the host intimal layer across the anastomosis because of a migration of native endothelial cells (43).

**Figure 7:**
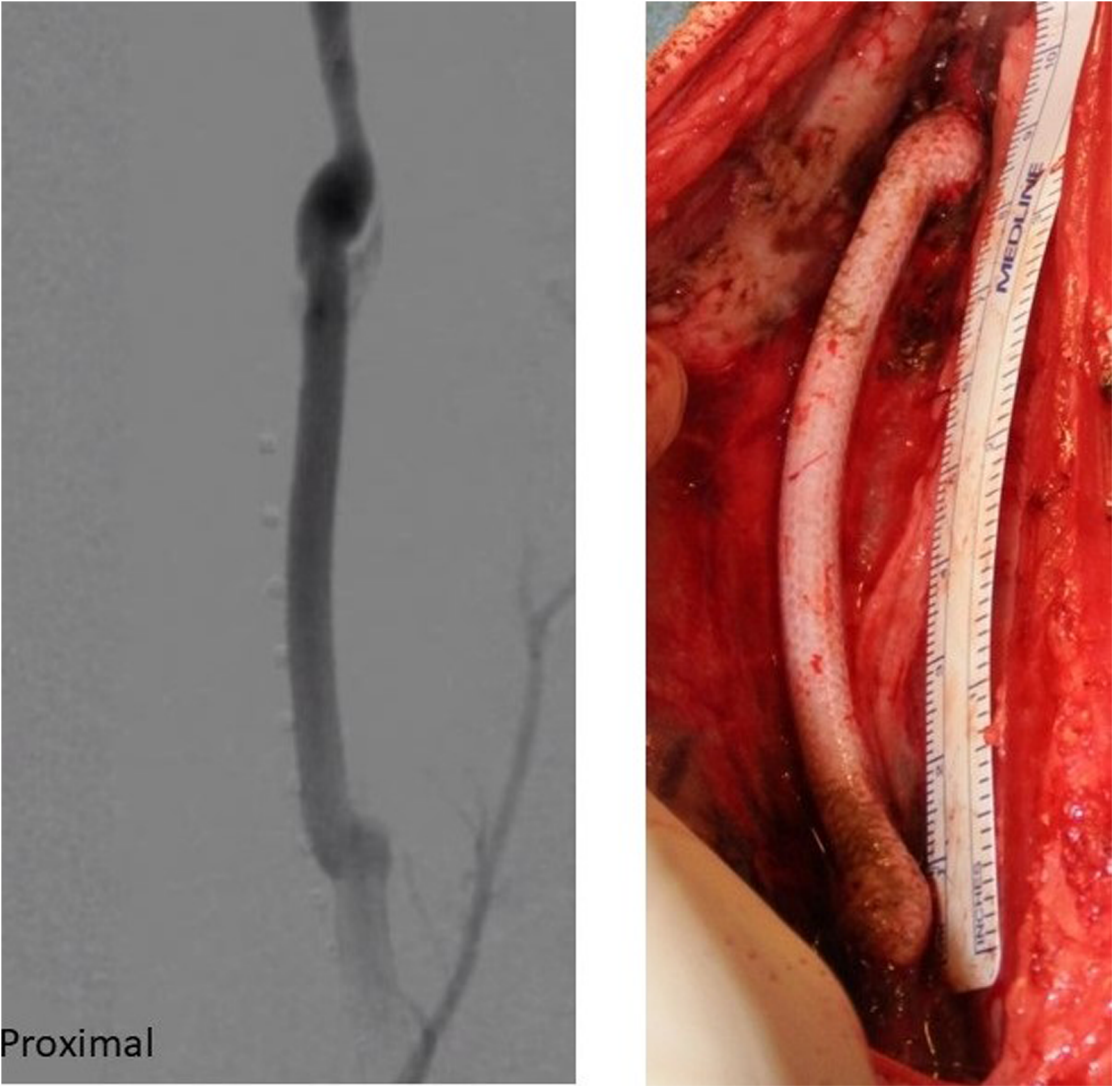
SF-SDVGs implanted in carotid artery of a sheep (**right**) and angiographic control revealing regular patency without dilatation or stenosis.

Other expectations include *in situ* biodegradation that facilitates constructive graft remodeling, mechanical resilience, and stability throughout the process of graft remodeling, the ability to attract and recruit host cells, and minimal immunogenicity (Figure 7).

Silk has a long history of being used as suturing material, largely due to its exceptional mechanical qualities. Various silk varieties have different amino acid contents, which results in native silk fibers having different mechanical strengths. As reported, native and regenerated forms of silk fibroin are both well used but they have different characteristics for different patterns. Native silk fibers confer better elasticity and slower time of degradation than regenerated, a crucial aspect to allow neo-tissue formation. The process of scaffolding as a silk vascular graft is dynamic and it is important to have a material with slower biodegradation time with higher strength and mechanical resistance. Regenerated forms are more managed to have a scaffold with quicker time of degradation as hydrogels, skin films or others not grafting use (44).

The spinning procedure and other industrial alterations that could be made to the biomaterial are additional influential aspects that impact mechanical qualities.

SF-based vascular grafts are excellent candidates for the engineering of products intended as “off-the-shelf” devices due to their promising biological responses. Since pre-seeding with cells is no longer necessary, this eliminates associated time delays and costs and minimizes the steps for graft preparation before implantation. Fibroin is a biodegradable protein derived from silk that provides an antithrombotic surface and serves as an ideal scaffold for various cell types in tissue engineering (45).

Modern technologies have demonstrated the capacity to logically implement the clinical viability of various SDVGs by providing results that are convincing and attest to the blooming nature of this subject. Despite these developments, none of the SDVGs have yet been commercialized, and only a small number of prospective targets are close to being translated into clinical practice. Existing research points to a few essential prerequisites for the clinical feasibility of SDVGs. The optimal solution would be widely available SDVGs, which would also need to be economically feasible and have sufficient mechanical and biological qualities.

Numerous healthcare items with silk as their substrate have been developed thanks to significant advancements in silk biomaterial research. Adipose, cartilage, bone, and wound dressing are just a few of the important non-vascular tissues that can be regenerated using products based on silk fibroin. Additionally, silk's prospective applications have been studied in the fields of medication delivery systems, medical implants, and occasionally evidenced by clinical proof. Its use has also been expanded to include the treatment of several dermatological disorders as well as breast and abdominal wall reconstruction.

In the vascular system an important topic for the evaluation of a new graft is the biocompatibility and haemocompatibility (44).

Usually, SF graft works as a scaffold for the progression of endothelial cells and progressive endothelialization of the substitute which could be the key point of its application. It is also reported the relationship between silk and human cells. Silk grafts showed to favor the adhesion, growth and survival of isolated human cells of three cell types of human peripheral arteries (endothelial cells, smooth-muscle cells and adventitial fibroblasts) respect to a polystyrene surface (46).

All the cell types, once cultured on either SilkGraft or polystyrene, exhibited similarly low basal levels of TIMP-2, a metalloprotease blocker which regulates extracellular matrix (ECM) remodeling processes and interactions between cells and ECM. It is interesting to note that no significant pro-inflammatory cytokines (Tumor Necrosis Factor-α (TNF-α), or of profibrotic cytokines (Transforming growth factor-b (TGF-b) were secreted by the three cell types grown on either substrate. The patterns of cytokines and chemokines secreted suggest a proliferative attitude while neatly excluding a pro-inflammatory and/or profibrogenic proclivity.

The biological haemocompatibility of SF is corroborate by lack complement activation and hemolysis meanwhile the cell adhesion, intense metabolic activities and the expansion of the cell populations cultured with no secretion of pro-inflammatory or pro-fibrotic cytokines show the high level of compatibility of SF rather than a synthetic graft (47) and this hemocompatibility of silk-based TEVGs has been validated in animal studies (48).

An important tool of Silk fibroin is the possibility to seed it with different cells. It has been reported that silk fibroin is a good scaffold for the cells of the endothelial wall but also bone marrow seeding or other cells type useful for the different use of the graft to reduce the thrombogenicity (49). The difference between seeded and not-seeded grafts is related to the use. Especially as vascular substitute, for the most, a not seeded graft is indicated, possibly made with multiple layers to reach more resistance and strength.

Although silk grafts have demonstrated extraordinary long-term regeneration capability in trials on small animals, their effectiveness in larger animals remains underdeveloped and field for future studies.

The development of other silk variants is still in its infancy and requires further attention. A couple of silk-based products that have been validated for clinical use belong to the tissue regeneration (41). While FDA permission has been obtained for the use of regenerated silk-based scaffolds in the reconstruction of non-vascular tissues, at the present, none of the silk-based SDVGs have reached the stage of human clinical trials yet.

Natural materials offer a solid blueprint to reinterpret, reengineer, and simplify modern manufacturing while preserving sustainability.

**Figure 8:**
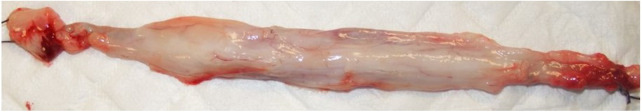
Anatomo-pathological sample of explanted SF-SDVG shows a complete healing of the graft and surrounding tissues.

SF is minimally immunogenic and avoid fibrous capsule encapsulation by chronic immune response. The factors which are determining the bio response of SF-based tissue-engineered grafts include the format of the material, implantation site and degradation rate and time. Hard tissue implantation and shorter degradation time would result in minimal immunogenicity, while SF matrices having longer degradation time and implanted at soft tissue sites would elicit comparatively elevated immunogenic response (50). This reaction could be considered as an advantage because the tissue is involved in constructive graft remodeling (51) (Figure 8).

A crucial aspect is the mechanic reaction and strength of the biodegradable materials to maintain a structure without degradation before a good vessel healing and organization avoiding rupture or aneurysm formation causing hemorrhagic complications. Gradual proteolytic degradation takes longer in silk: silk fibers generally lose most of their tensile strength less or equal to 1 year *in vivo* and become unrecognized at the site less or equal to 2 years (52).

Last important topic for SF is the innovative formats of 3D printing for biological research and engineering technology. The capacity of SF to be processed into a variety of configurations for various applications provides as an additional benefit over other polymers. Its use in many applications is made possible by a tunable degradation pattern and exceptional physical and chemical characteristics. Due to the existence of additional desirable qualities, composite materials of SF and blends have emerged as a superior alternative option for many clinical and biological applications. There are no reports about the joining of SF and 3D prints in vascular tissues (in other fields this technique is already developed), but considering the opportunities of both, in the next future an increase of the possibilities of revascularization could be really evaluated (53).

In our review all preclinical studies were analyzed. If a new material for vascular grafts should be studied, the way to the clinical phase is surely longer than other districts especially if it is a scaffold for the cellular growing. Literature reports some good results of pre-clinical phase and good efficacy of the graft as vascular scaffold for arterial, venous and artero-venous reimplantation. Clinical phase requires a particular assurance of the strength and resistance of the graft especially in the anastomotic sites. Results from different experiences are encouraging but we need more data from *in vivo* in large animal models before trying the silk fibroin grafts in humans (54, 55).

## 6. Conclusions

Our review showed how vascular grafts encompassing silk fibroin are promising for further biological development in animals and humans. Biodegradability and biocompatibility of this natural polymer will likely foster the development as biomaterial of choice for vascular reconstruction.

The key point remains that future studies should investigate silk-based vascular grafts in preclinical large animal models, focusing on the optimization of graft design parameters. Since the exact mechanism of graft remodeling and neo-tissue formation is not clear considering the heterogeneity of studies involving SF vascular grafts, further insight into the endothelialization of the grafts must be provided, along with long term outcomes on thrombogenicity and patency rates. Inevitably, the coming of age of SF as predilected biomaterial for vascular reconstruction will be sanctioned by the first clinical trial in humans.
